# Theranostic Properties of a Survivin-Directed Molecular Beacon in Human Melanoma Cells

**DOI:** 10.1371/journal.pone.0114588

**Published:** 2014-12-11

**Authors:** Sara Carpi, Stefano Fogli, Ambra Giannetti, Barbara Adinolfi, Sara Tombelli, Eleonora Da Pozzo, Alessia Vanni, Enrica Martinotti, Claudia Martini, Maria Cristina Breschi, Mario Pellegrino, Paola Nieri, Francesco Baldini

**Affiliations:** 1 Department of Pharmacy, University of Pisa, Pisa, Italy; 2 Institute of Applied Physics “Nello Carrara,” IFAC-CNR, Sesto Fiorentino, Florence, Italy; 3 Department of Translational Research and New Technologies in Medicine and Surgery, University of Pisa, Pisa, Italy; Thomas Jefferson University, United States of America

## Abstract

Survivin is an inhibitor of apoptosis overexpressed in different types of tumors and undetectable in most terminally differentiated normal tissues. In the current study, we sought to evaluate the *in vitro* theranostic properties of a molecular beacon-oligodeoxynucleotide (MB) that targets survivin mRNA. We used laser scanning confocal microscopy to study MB delivery in living cells and real-time PCR and western blot to assess selective survivin-targeting in human malignant melanoma cells. We further assess the pro-apoptotic effect of MB by measuring internucleosomal DNA fragmentation, dissipation of mitochondrial membrane potential (MMP) and changes in nuclear morphology. Transfection of MB into A375 and 501 Mel cells generated high signal intensity from the cytoplasm, while no signal was detected in the extracellular environment and in survivin-negative cells (i.e., human melanocytes and monocytes). MB time dependently decreased survivin mRNA and protein expression in melanoma cells with the maximum effect reached at 72 h. Treatment of melanoma cells with MB induced apoptosis by significant changes in MMP, accumulation of histone-complexed DNA fragments in the cytoplasm and nuclear condensation. MB also enhanced the pro-apoptotic effect of standard chemotherapeutic drugs tested at clinically relevant concentrations. The MB tested in the current study conjugates the ability of imaging with the pharmacological silencing activity against survivin mRNA in human melanoma cells and may represent an innovative approach for cancer diagnosis and treatment.

## Introduction

Cutaneous malignant melanoma is a type of skin cancer that develops in response to genetic and environmental pressures leading to oncogenic transformation of normal human melanocytes. Currently, more than one hundred thousand melanoma skin cancers occur globally each year [1]. Treatment of melanoma by surgical excision is usually curative only when the tumor is detected early [2], while survival is poor for patients with advanced disease [3]. This poor prognosis largely results from resistance to conventional chemotherapy [4] and the lack of effective treatments that may modify the natural history of the disease [5].

Survivin is a member of the inhibitor of apoptosis (IAP) family [6] that plays a key role in the regulation of cell division, apoptosis, cell migration and metastasis [7]–[9]. Furthermore, survivin is involved in the promotion of angiogenesis and chemoresistance [9]. Unlike other anti-apoptotic proteins, survivin is undetectable in most terminally differentiated normal tissues, while it is overexpressed in human cancer [10]. For instance, survivin is highly overexpressed in malignant melanoma cells compared with normal melanocytic nevi and normal differentiated skin tissues [11]. Furthermore, a retrospective analysis performed in melanoma patients has revealed that survivin up-regulation is correlated with decreased survival rate, increased relapse, and chemoresistance occurrence [12]–[14]. These features make survivin a promising target against which novel anti-cancer drugs could be developed.

It is widely recognised that therapeutic strategies targeting survivin (e.g., antisense oligonucleotides and siRNA) can induce tumor cell death, circumvent drug resistance and sensitize cancer cells to chemotherapeutic drugs [9]. More recently, much attention has been directed to molecular beacons (MBs) as potential theranostic agents [15]. MBs are stem-loop-folded oligodeoxyribonucleotides with fluorophore and quencher dyes conjugated to the opposite ends of the hairpin. In the absence of the complementary nucleic acid target (mRNA, in the case of this work), the fluorescence of the fluorophore is quenched by the closely located quencher [16]. Otherwise, the hybridization with target nucleic acid opens the hairpin, generates probe-analyte duplex that physically separates the fluorophore from quencher, allowing a fluorescence signal to be emitted upon excitation [17], [18].

It has been reported that MB technology may allow assessing gene expression *in vivo* with high sensitivity and low background signal and may help to noninvasively detect cancer in its early stages. Furthermore, MB can also produce therapeutic effect by binding and down-regulating its target gene [19]. Several lines of evidence provided by the antisense therapy research, demonstrate that complementary pairing of a short segment of an exogenous oligonucleotide to mRNA can have a profound impact on protein expression levels and even cell fate. In fact, binding of MB to mRNA can trigger RNase H mediated mRNA degradation [17].

In the current study, we provide evidence of the simultaneous imaging and pro-apoptotic activities of a previously described MB [20] and demonstrate that such effects are associated with selective targeting of survivin mRNA in human melanoma cells. This approach may represent an innovative strategy for the development of novel theranostic drugs in human cancer.

## Materials and Methods

### Cell cultures

The human malignant melanoma A375 cell line (American Type Culture Collection, Rockville, MD, USA) was cultured at 37°C in a humidified atmosphere containing 5% CO_2_ in DMEM supplemented with l-glutamine (2 mM), 10% heat-inactivated fetal bovine serum (FBS) and 1% (w/v) penicillin/streptomycin (Sigma-Aldrich, Milan, Italy). The human metastatic melanoma 501 Mel cell line was a kind gift from Dr. Poliseno (Oncogenomics Unit, Core Research Laboratory, Istituto Toscano Tumori c/o IFC-CNR, Pisa, Italy). 501 Mel cells were cultured in the same condition of A375 with glucose supplement. Human bronchial smooth muscle cells (BSMC; Lonza, Walkersville, MD, USA) were maintained exactly as recommended by the manufacturer in an optimized medium containing 5% fetal bovine serum, 5.5 mM glucose, 50 µg/ml gentamicin, 50 ng/ml amphotericin-B, 5 ng/ml insulin, 2 ng/ml basic fibroblast growth factor and 0.5 ng/ml epidermal growth factor (SmGM-2 Bullet Kit, Lonza). Human melanocytes (PromoCell GmbH, Germany) were cultured at 37°C in a humidified atmosphere containing 5% CO_2_ in Melanocyte Growth Medium M2 (PromoCell GmbH, Germany). Human monocytes were a kind gift from Dr. Celi (Department of Surgery, Medical, Molecular, and Critical Area Pathology). Monocyte isolation was performed as described previously [21]. The procedure was approved by the local ethics committee at the University of Pisa and was in accordance with the Declaration of Helsinki. A signed consent was obtained from all donors.

### Drugs

Docetaxel (DTX) and Cisplatin (CisPt) were purchased from Sigma–Aldrich, Milan, Italy. DTX was dissolved in dimethyl sulfoxide (DMSO) and diluted with culture medium (DMSO final concentration of 0.0001%, v/v), while CisPt was dissolved in water.

### Transfection

Cells were transfected with 100 nM antisense oligodeoxynucleotides using Lipofectamine 2000 (Ref. 11668-027, Invitrogen Life Technologies, Carlsbad, CA, USA), which has been reported to yield high transfection efficiency (≈70%) in A375 cells [22]. A molecular beacon (MB), which targets nucleotides 149–163 of survivin mRNA, and the other oligodeoxynucleotides were synthesized by IBA (Göttingen, Germany) [20]. ATTO647N (λ_abs_ 644 nm, λ_em_ 669 nm) and Blackberry Quencher 650 (λ_max_∼650 nm, useful absorbance between 550 and 750 nm) were used as fluorophore/quencher pair. A fluorescent DNA probe, with the same oligonucleotide sequence of MB but not structured as a molecular beacon and labelled only with the ATTO647N dye, was used as control. Oligonucleotide sequences are listed in Table 1.

**Table 1:** Oligonucleotide sequences of Molecular Beacon (MB) and probe.

Name	Sequence
MB	5′-ATTO647N-CGACGGAGAAAGGGCTGCCACG/thiol-(C6)-dT/CG–BBQ-3′
Probe	5′-ATTO647N-GAGAAAGGGCTGCCA/thiol-3′

BBQ: BlackBerry Quencher; ATTO647N: fluorescent label (report). Underlined bases are the ones forming the loop.

### *In vitro* confocal microscopy in living cells

Laser scanning confocal microscopy was carried out by using a microscope RadiacePLUS (Bio-Rad). Briefly, cells were plated on 35 mm µ-dishes (Ibidi Giemme Snc, Milan, Italy) and treated with 100 nM antisense oligodeoxynucleotides (MB or probe). All observations were done with an oil immersion x 40 Nikon objective lens (NA = 1.3). Fluorescence was evaluated at different time points during the transfection (excitation and emission wavelengths of 644 and 669 nm, respectively). Acquired images were processed using the open source Image-J software.

### RT-PCR and quantitative real-time PCR analyses

Total RNA from cells was extracted by using the RNeasy Mini kit, following manufacturer's instructions, and reverse-transcribed by the QuantiTect Reverse Transcription kit (Qiagen, Valencia, CA, USA). RT-PCRs were performed by the HotStartTaq Master Mix kit (Qiagen, Valencia, CA, USA). Primer sequences are listed in Table 2. Thermal cycle conditions were as follows: 95°C for 15 min, 35 cycles of denaturation at 95°C for 1 min followed by annealing and extension at 72°C for 1 and 10 min, respectively. Detection of the RT-PCR products was performed by agarose gel electrophoresis and ethidium bromide staining.

**Table 2:** Primer nucleotide sequences, Ta and amplicon length used for PCR experiments.

Name	Primer nucleotide sequences	Ta (°C)	Amplicon lenght
GAPDH	5′-GTGAAGGTCGGAGTCAACG- 3′ (F)	59	301 pb
	5′GGTGAAGACGGCCAGTGGACT- 3′ (R)		
Beta-actin	5′- AACTGGAACGGTGAAGGTGAC -3′ (F)	61	138 pb
	5′- GACTTCCTGTAACAACGCATCTC - 3′ (R)		
Survivin	5′- ACCAGGTGAGAAGTGAGGGA -3′ (F)	59	309 pb
	5′- AACAGTAGAGGAGCCAGGGA - 3′ (R)		

Real-time PCR was performed with SsoFast Eva Green Supermix (Ref. 172-5201, Bio-Rad, CA, USA). Samples were amplified using the following thermal profile: 95°C for 30 s, 40 cycles of denaturation at 95°C 15 s followed by annealing for 30 s and 72°C for 30 s, with a final step at 65°C for 5 s. *GAPDH* and *β-actin* were used as housekeeping genes.

### Western blot analysis

Cell lysates were collected after treatments at different time points. Samples containing the same amount of protein (40 µg) were separated on a 15% SDS-polyacrylamide gel electrophoresis, transferred to a nitrocellulose membrane (Sigma–Aldrich, Milan, Italy), blocked with 5% non-fat milk in TBE and probed with specific antibodies. Incubation was performed at 4°C overnight with anti-survivin (Ref. sc-17779, Santa Cruz Biotechnology, Santa Cruz, CA, USA) and anti-β-actin (Ref. A2228, Sigma–Aldrich, Milan, Italy) antibodies. Membranes were then washed with blocking solution and incubated with secondary antibodies conjugated with horseradish peroxidase (Ref. sc-2005, Santa Cruz Biotechnology, Santa Cruz, CA, USA). Chemiluminescence detection was performed using Western Blotting Luminol Reagent (Ref. sc-2048, Santa Cruz Biotechnology, Santa Cruz, USA), according to the manufacturer's instructions. Quantification of proteins on SDS-PAGE gels was performed using ImageJ densitometry software and signal intensities were normalized to those for β-actin.

### Internucleosomal DNA fragmentation

The Cell Death Detection ELISA Kit (Ref. 11774452001, Roche, Mannheim, Germany) was used for assessing apoptosis in A375 cells treated with MB, according to the manufacturer's protocol. Briefly, A375 cells were treated with 100 nM MB at different times. After treatment, 10^4^ cells were lysed and centrifuged at 200×g for 10 min and the supernatant placed into a streptavidin-coated microplate. A mixture of anti-histone-biotin and anti-DNA-POD was added and incubated at room temperature for 2 h. After incubation, unbound antibodies were removed from the solution and the nucleosomes were quantified by colour development with substrate. Optical density was measured at 405 nm with the Infinite M200 NanoQuant instrument (Tecan, Salzburg, Austria).

### Mitochondrial membrane potential (ΔΨm)

Changes in mitochondrial membrane potential (ΔΨm) during the early stages of apoptosis were assayed using the Muse MitoPotential assay (Ref. MCH 100110, Merck Millipore; Darmstadt, Germany) in A375 cells treated with MB alone or in combination with Cis-Pt or DTX. Briefly, cells were harvested and the cell pellet was suspended in assay buffer (10^5^ cells/100 µl). MitoPotential dye working solution was added and the cell suspension incubated at 37°C for 20 min. After the addition of Muse MitoPotential 7-AAD dye (propidium iodide) and incubation for 5 min, changes in ΔΨm and in cellular plasma membrane permeabilization were assessed using the fluorescence intensities of both the dyes analysed by flow cytometry (Muse Cell Analyzer, Merck Millipore; Darmstadt, Germany).

### Determination of nuclear morphology

Changes in nuclear morphology were assessed after transfection with MB and in lipofectamine-treated cells for 48 and 72 h. Cells were fixed with 4% paraformaldehyde on 8-well chamber slides. After washing with PBS, cells were incubated with DAPI 300 nM (Invitrogen Life Technologies, Carlsbad, CA, USA). Life fluorescence analysis was realized with Eclipse E600FN Nikon microscope. The fluorophore DAPI is a fluorescent label for the blue spectral region (excitation and emission wavelengths of 360 and 460 nm, respectively) (magnification 40×WD).

### Statistical analysis

All experiments were performed in triplicate and results were analyzed by GraphPad Prism 5 (GraphPad Software, San Diego, CA, USA). Data were shown as mean values ± standard error of the mean (SEM) obtained from at least three independent experiments. Statistical analyses were performed by Student's *t*-test or one-way ANOVA followed by the Bonferroni's multiple comparison test.

## Results

### Survivin mRNA expression in different cell types

**Figure 1:**
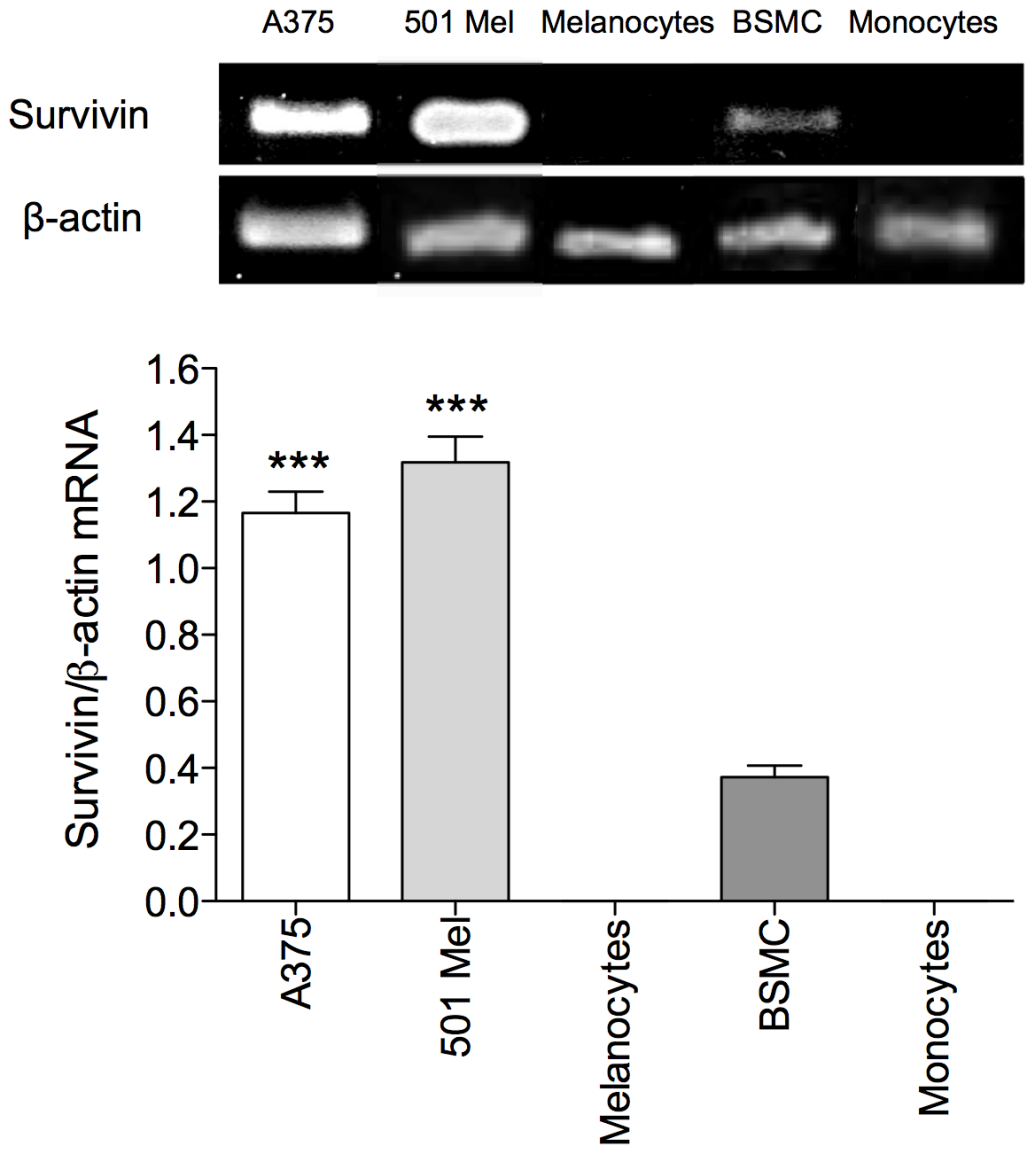
*Survivin* gene espression in A375, 501 Mel, melanocytes, BSMC, and monocytes. Values were expressed as mean ± standard error of the mean (SEM) from three separate experiments. ***p<0.001, compared with melanocytes (ANOVA followed by the Bonferroni's multiple comparison test).

RT-PCR experiments followed by densitometry and quantification of electrophoresis bands clearly demonstrated that survivin was differentially expressed in human melanoma cells compared to normal cells. Specifically, survivin mRNA levels were significantly higher in A375 and 501 Mel cells than in BSMC (p<0.001), while human melanocytes and monocytes did not express survivin (Fig. 1). β-actin was used as housekeeping gene because its expression levels did not significantly change in all of our experimental conditions.

### MB specific binding to survivin mRNA

**Figure 2:**
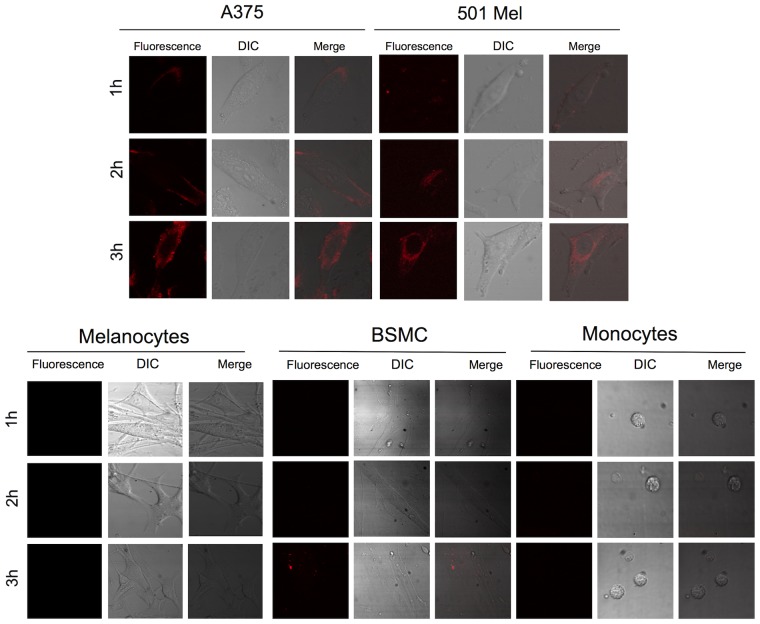
Confocal microscopy images of survivin expression in the cytoplasm of living cells. Cells were treated with 100 nM MB (red) and fluorescence was measured at 1, 2 and 3 h following start of transfection. Confocal fluorescent images are shown in the left column, transmission DIC images in the central column, while the merged images are also shown in the right column. The fluorescence images were obtained by using excitation and emission wavelengths of 645 and 669 nm, respectively and an oil immersion objective (x40, NA = 1.3).

*In vitro* bioimaging was used to assess transfection of the MB via Lipofectamine into different cell types tested and its target-specific structure opening with fluorescence emission. Confocal microscopy of living cells clearly demonstrated that MB transfection generated a fluorescence signal according to survivin expression levels and time of exposure. Specifically, confocal microscopic images of A375 and 501 Mel cells showed high signal intensity in these specific cell types (Fig. 2) indicating that MB could cross the cell membrane, bind to survivin mRNA and generate high fluorescence emission. Otherwise, the fluorescence signal was significantly lower in the case of BSMC cells, while no fluorescence could be detected in human melanocytes and monocytes (Fig. 2). Noteworthy, an intense red fluorescent signal was observed (data not shown) when human monocytes were transfected with the fluorescent probe. This demonstrated that Lipofectamine was able to transfect both melanoma cells and normal cells (i.e., melanocytes and monocytes), and that the MB specifically opened with fluorescence emission only in survivin mRNA positive cells (i.e., A375 and 501 Mel cells). Specificity of the bases forming the MB loop for survivin versus other members of the IAP family was confirmed by BLAST analysis.

### MB downregulation of survivin expression and protein synthesis

**Figure 3:**
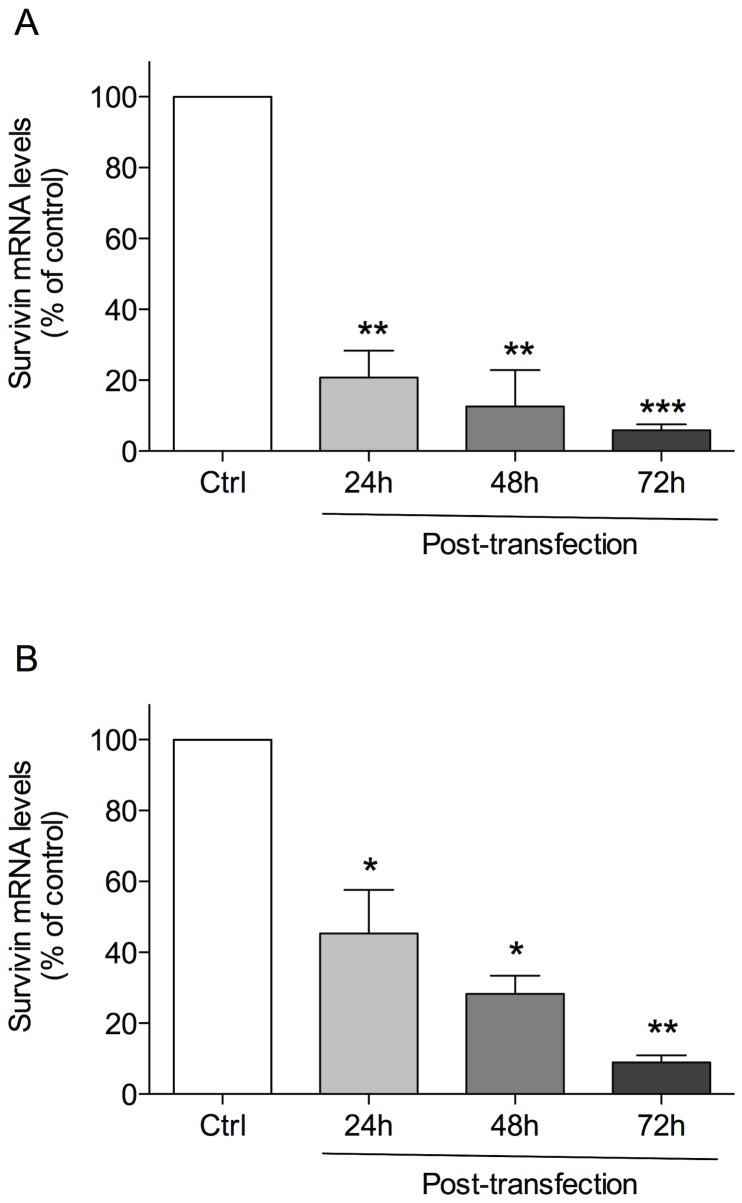
Real-time PCR assessment of *survivin* gene expression in (A) A375, and (B) 501 Mel cells. Cells were transfected with lipofectamine-MB or treated with lipofectamine alone (Ctrl) and analyzed after 24, 48 and 72 h. Values were expressed as mean ± standard of the mean (SEM) from three separate experiments. *p<0.05, **p<0.01, ***p<0.001, (ANOVA followed by the Bonferroni's multiple comparison test).

We performed real-time PCR experiments to quantitatively assess the effect of MB on *survivin* gene expression in different cell types. Treatment with MB for 24 h significantly decreased *survivin* expression by 79.3±7.6% (p<0.01), as compared to cells transfected with control (only lipofectamine) in A375 cells (Fig. 3A). Prolonged cellular exposure to MB further decreased expression of *survivin* and the maximum effect was reached after 72 h (-94±1.6%, as compared to control). These findings were also confirmed in 501 Mel cells in the same experimental conditions (Fig. 3B).

**Figure 4:**
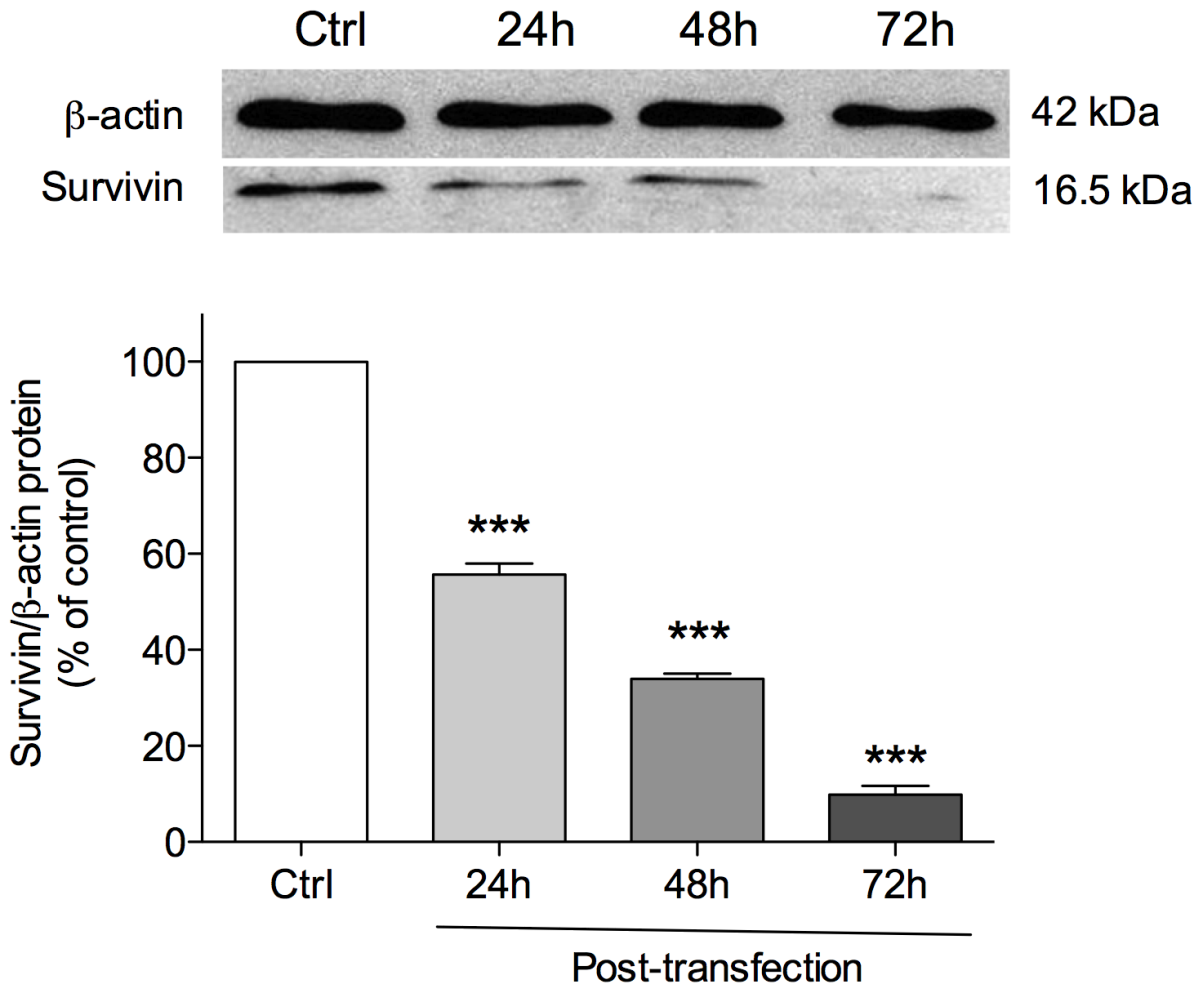
Detection of survivin protein levels by Western Blot analysis in A375 cells. Cells were transfected with lipofectamine-MB or treated with lipofectamine alone (Ctrl) and analyzed after 24, 48 and 72 h. Values were expressed as mean ± standard of the mean (SEM) from three separate experiments. ***p<0.001, (ANOVA followed by the Bonferroni's multiple comparison test).

Expression levels of survivin protein in A375 cells was assessed by western blot and the bands on the blot image were analysed by densitometry. In A375 lysates, the survivin antibody mainly labelled a band corresponding to an apparent molecular weight of 16.5 KDa. Cell treatment with MB time-dependently inhibited survivin protein expression by 44.3±2.4, 66.1±1.1, and 90.1±1.8% after 24, 48 and 72 h, respectively, as compared to control (Fig. 4).

### MB induction of apoptosis

The ability of MB to induce apoptosis in human melanoma cells was investigated by evaluating dissipation of mitochondrial membrane potential (ΔΨm), internucleosomal DNA fragmentation, and nuclear morphological changes.

**Figure 5:**
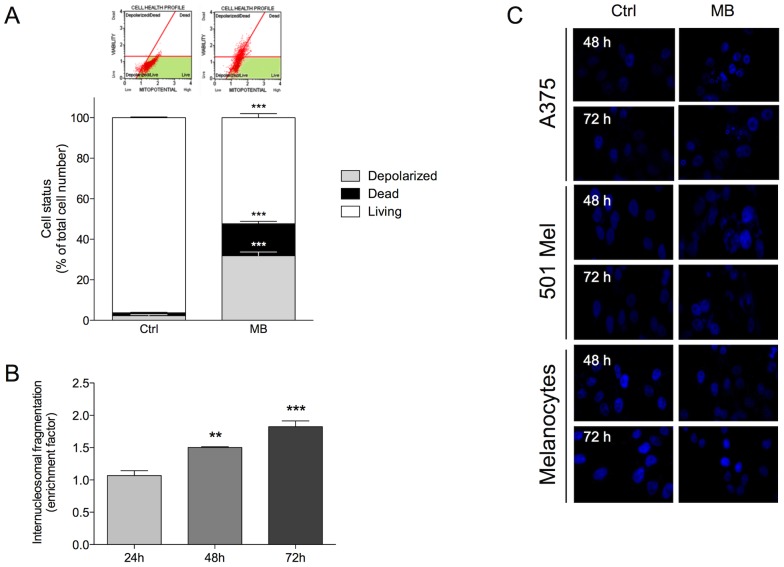
(A) Dissipation of the mitochondrial membrane potential (ΔΨm) in A375 cells at 48 h. A representative dot plots in live, depolarized/live, depolarized/dead and dead phase is showed in the upper panel, whereas the mean percentages of the total cell number for each cell status are reported in the lower panel. Values were expressed as mean ± standard of the mean (SEM) from three separate experiments. ***p<0.001, as compared to control (Student's *t*-test). (B) Time-dependent fragmentation of internucleosomal DNA in A375 cells after MB treatment at 100 nM for 24, 48 and 72 h. Values were expressed as mean ± standard of the mean (SEM) from three separate experiments. *p<0.05, **p<0.01, as compared to 24 h (ANOVA followed by the Bonferroni's multiple comparison test). (C) Nuclear morphology revealed by DAPI staining of A375, 501 Mel cells and human melanocytes transfected with MB or treated with lipofectamine alone (Ctrl) at 48 and 72 h.

Treatment with MB for 48 h induced a significant (p<0.001) variation in ΔΨm in about 35% of total cells in A375, suggesting the involvement of the intrinsic pathway in the molecular mechanism of MB action. Otherwise, control experiments demonstrated the absence of significant changes in ΔΨm (Fig. 5A). A representative dot plots in live, depolarized/live, depolarized/dead and dead phase is showed in the upper panel in Fig. 5A.

MB induced accumulation of histone-complexed DNA fragments in the cytoplasmic fraction of A375 cell lysates after 48 and 72 h, while after 24 h it did not significantly induce cell death (Fig. 5B). This is probably due to the fact that major DNA fragmentation is a late event in apoptosis. According to this, DAPI staining experiments showed that many A375 and 501 Mel cells exposed to MB for 48 and 72 h exhibited nuclear condensation and chromatin fragmentation, while human melanocytes did not (Fig. 5C). Noteworthy, increased formation of multinucleated cells was observed in MB-treated cells, whereas control cells showed normal nuclear morphology (data not shown).

### MB enhancement of chemotherapy-induced apoptosis

**Figure 6:**
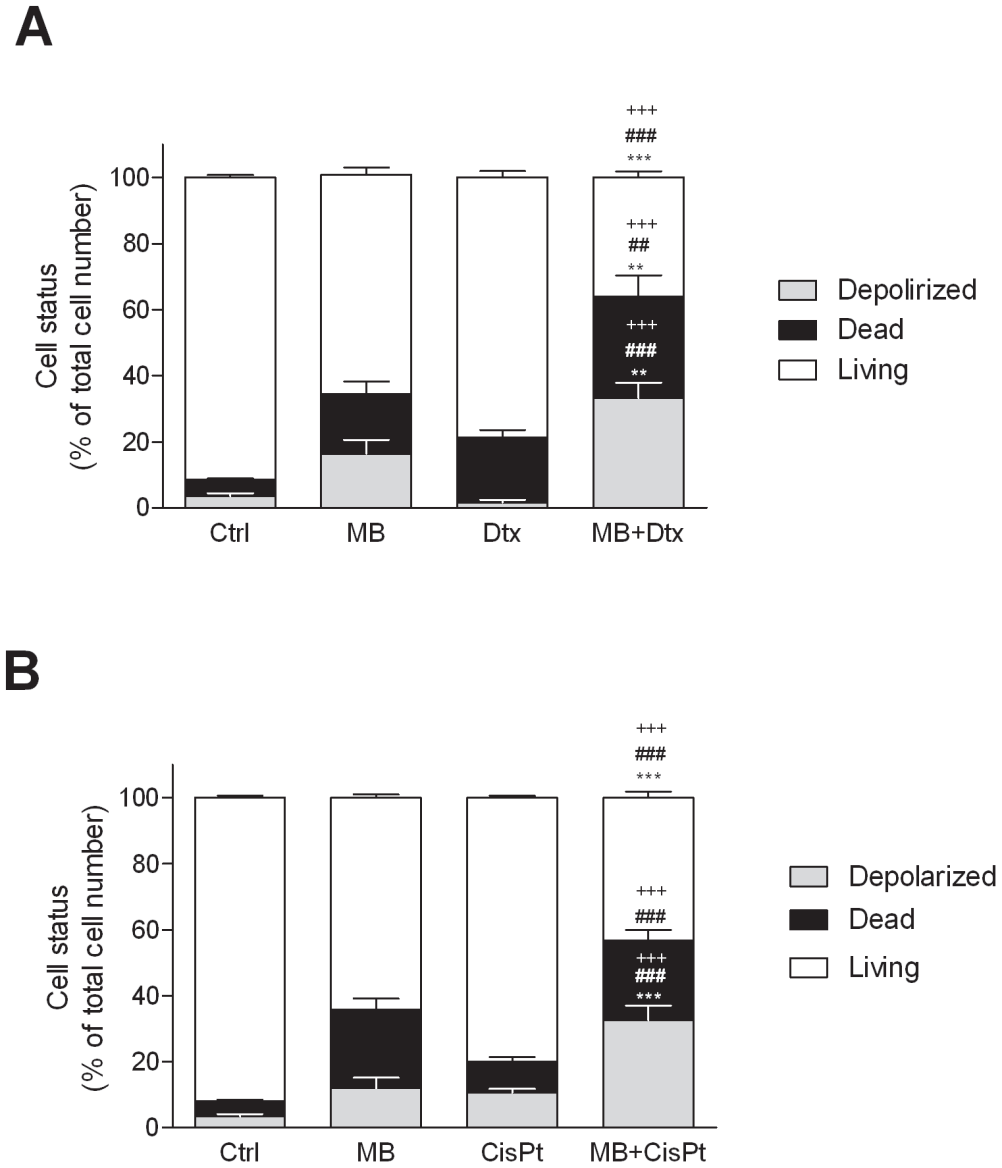
Dissipation of the mitochondrial membrane potential (ΔΨm) induced by MB, cisplatin (CisPt) and docetaxel (DTX), alone or in combination schedules, in A375 cells. Cells were preincubated with MB at 100 nM for 48 h in the presence or absence of CisPt at 1 µM (A) or DTX at 10 nM (B) for 24 h. Values were expressed as mean ± standard of the mean (SEM) from three separate experiments. *p<0.05, **p<0.01, compared with MB alone; ##p<0.01, ###p<0.001, compared with CisPt or DTX alone; +++p<0.001, compared with control (Ctrl), (ANOVA followed by the Bonferroni's multiple comparison test).

To assess the ability of MB to increase the proapoptotic effects induced by DTX and CisPt, A375 cells were pre-incubated with MB at 100 nM for 48 h, followed by DTX at 10 nM or CisPt at 1 µM for 24 h. Apoptosis after single agent or combination treatments was analyzed by measuring ΔΨm. The combination of MB with DTX or CisPt at low concentrations produced an effect greater than that of each drug alone (Fig. 6). Specifically, the percentage mean values of depolarized A375 cells after single DTX and MB treatments were 1.64±0.72% and 16.34±4.24%, respectively, as compared to control. It is worth mentioning that MB *plus* DTX significantly (p<0.001) increased the percentage of depolarized cells up to 33.28±4.59%, compared to control (Fig. 6A). The statistical analysis also highlighted a significant (p<0.01) ΔΨm increase in combination *versus* single agent treatments. In a similar manner, the percentage of depolarized cells after treatment with single CisPt and MB was 10.64±0.92% and 11.93±2.76%, respectively, whereas it was of 29.98±4.09% after combination treatment (Fig. 6B).

## Discussion

**Figure 7:**
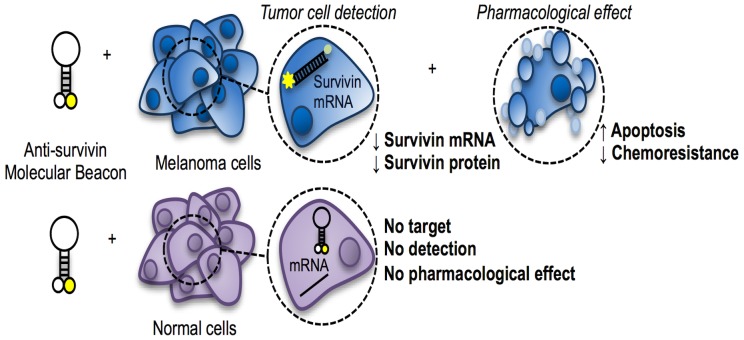
Overview of theranostic properties of anti-survivin moleular beacon.

Although depletion of survivin by siRNA has been previously reported to increase the sensitivity of cancer cells to chemotherapy *in vivo* [23], [24], siRNA-based therapeutics targeting survivin *per se* does not have the potential to detect cancer cells. Our findings provide evidence of a novel potential strategy for both cancer diagnosis and treatment. Specifically, we deeply characterized *in vitro* a MB that conjugates the ability of imaging of survivin with the pharmacological silencing activity in human melanoma cells (Fig. 7). The ability of the MB-lipofectamine system to cross the cell membrane and to penetrate into the cytoplasm of A375 and 501 Mel cells was clearly demonstrated in the current study by confocal microscopy. Transfection of MB into human melanoma cells generated high signal intensity from the cytoplasm, while no signal was detected in the extracellular environment or in survivin-negative cells (i.e., human melanocytes and monocytes). These findings demonstrate the diagnostic capability of the MB that may have great potential to translate into clinical applications. In this regards, a tumor-specific accumulation in the *in vivo* setting could be reached *via* nanoparticle-delivered transfection, therefore permitting enhanced permeability and retention effect in cancer tissues [25].

It is widely recognized that survivin expression in melanoma is inversely correlated with patient survival [26]. Furthermore, high survivin expression is associated with resistance to chemotherapeutic agents [8] and survivin-overexpressing melanoma cells have been reported to have a potential role in lung metastasis [27], [28]. Therefore, the development of targeted contrast agents (such as fluorescent probes) coupled with optical imaging techniques could allow to characterize tumor progression *in vivo* with great sensitivity and selectivity.

The current study demonstrated the therapeutic potential of the tested MB. This MB was already described in literature [20] for the detection and quantification of survivin mRNA. However, no evidence was provided on its silencing activity in pancreatic cancer cells [20], [29], whereas other works showed that a partially homologous MB did not induce any variation in survivin expression in human breast cancer cells [30].

Concerning the molecular beacon tested in this work, we found that treatment with MB markedly decreased survivin mRNA and protein expression associated with activation of apoptosis. In line with evidence highlighting the central role of mitochondrial survivin in the apoptotic machinery [31], we demonstrated that MB, at nanomolar concentrations, induced a significant loss of mitochondrial transmembrane potential in A375 cells. Furthermore, the increased formation of multinucleated cells observed in the current study after transfection with MB is also consistent with the critical role of survivin in the regulation of cytokinesis [32], [33]. Indeed, survivin is a component of the chromosomal passenger complex, which plays a role in chromosome condensation, interaction between kinetochores and microtubules at metaphase and midzone microtubule organization at anaphase [33].

Concerning the mechanism of action, MB is a stem-loop hairpin-structured oligonucleotide that works as an antisense oligonucleotide. In agreement with this notion, selective inhibition of survivin expression by the antisense oligonucleotide LY2181308 induced apoptosis, cell cycle arrest in the G2-M phase, and formation of multinucleated cells [34].

Another interesting point that needs to be discussed regards the role of survivin in chemoresistance [35]. For example, it has been reported that both docetaxel and cisplatin induced accumulation of survivin in different types of tumors including melanoma [36], [37], lung [38], breast [39], and gastric [40], [41] cancer. We have found that *in vitro* combination treatment of MB and docetaxel or cisplatin induced a greater rate of apoptosis than the sum of the single-treatment rates in A375 cells, suggesting that targeting survivin has the potential to increase the sensitivity of cancer cells to chemotherapeutics. The effect induced by combination schedules appears to be additive because it is approximately the sum of the percentage obtained from single treatments, both in terms of depolarized cells (i.e., cells committed to die) and dead cells. Moreover, it is also possible that survivin silencing may restore sensitivity to targeted therapy. In line with this notion, it has been recently demonstrated that blood survivin mRNA positivity was strongly related to a poor treatment outcome of EGFR-tyrosine kinase inhibitors in non-small cell lung cancer patients [42].

Although general transfection techniques (including those employing liposomes or dendrimers) might result in false positive signals, we demonstrated that cellular lipofection of tested MB was specific with no false positive results. Indeed, a bright red fluorescent signal was observed when cells that did not express survivin (i.e., monocytes) were transfected with the linear probe (the same fluorescent antisense oligonucleotide of MB lacking the quencher molecule), while no signal was detected after MB transfection.

Taken together, our findings provide evidence of a novel potential anticancer strategy for the simultaneous imaging and targeted therapy in human cutaneous melanoma. The ability to image specific RNAs *in vivo* may offer new opportunities in terms of development of clinical diagnostic procedures for the early detection of cancer, pharmacological monitoring of silencing effect, and follow-up after surgery or chemotherapy treatment.
